# Long-Term Outcomes of Rheohaemapheresis in the Treatment of Dry Form of Age-Related Macular Degeneration

**DOI:** 10.1155/2013/135798

**Published:** 2013-12-18

**Authors:** Jan Studnička, Eva Rencová, Milan Bláha, Pavel Rozsíval, Miriam Lánská, Vladimír Bláha, Jan Němčanský, Hana Langrová

**Affiliations:** ^1^Department of Ophthalmology, Charles University, Medical Faculty and Faculty Hospital, Hradec Králové, Czech Republic; ^2^Second Department of Internal Medicine, Haematology, Charles University, Medical Faculty and Faculty Hospital, Hradec Králové, Czech Republic; ^3^Department of Gerontology and Metabolic Care, Charles University, Medical Faculty and Faculty Hospital, Hradec Králové, Czech Republic; ^4^Department of Ophthalmology, University Hospital, Ostrava, Czech Republic

## Abstract

*Purpose*. Determining long-term effects of rheohaemapheresis on the dry form of age-related macular degeneration. *Methods*. This study evaluates 19 patients, average age of 67.6 years, treated with rheohaemapheresis and 18 patients, average age of 72.8 years, comprising the control group. Minimum follow up period was 3.5 years. Each treated patient received a series of 8 sessions of rheohaemapheresis of 1.5 plasma volumes within 10 weeks. We measured the drusenoid pigment epithelium detachment (DPED), best-corrected visual acuity (BCVA), electroretinography (ERG), and rheological parameters. *Results*. In the treatment group, the baseline BCVA was 0.74 (0.36–1.0) 95% CI and BCVA after 3.5 years was 0.79 (0.41–1.0) 95% CI (*P* = 0.726). In the control group, the baseline BCVA was 0.71 (0.15–1.0) 95% CI and BCVA after 3.5 years decreased to 0.7 (0.32–0.87) 95% CI (*P* = 0.031). Baseline DPED was 6.78 ± 3.79 mm^2^; after 3.5 years, it decreased to 4.13 ± 3.84 mm^2^ (*P* < 0.001). In the control group, the baseline DPED was 4.09 ± 3.48 mm^2^; after 3.5 years, it increased to 6.69 ± 4.2 mm^2^ (*P* = 0.001). We noted increasing levels of positive wave peaking at 50 milliseconds (P50) after treatment (*P* = 0.022) and a stable amplitude of photopic responses of treated patients. *Conclusion*. Over the long term, rheohaemapheresis reduced the DPED, improved the function of photoreceptors, and prevented the decline of BCVA.

## 1. Introduction

Age-related macular degeneration (AMD) is a degenerative retinal disease which causes significant structural changes in the macular region. It is the most common cause of practical blindness in people over 65 years of age in developed countries [1]. The majority of patients present with a slowly developing “dry” atrophic form of AMD, but about 10% of patients will develop a rapidly progressing “wet” form with choroidal neovascular membrane (CNV), which is responsible for loss of vision in most patients [2]. AMD usually starts as a dry form with soft drusen/distinct soft drusen >63 *μ*m, indistinct soft drusen >125 *μ*m, or reticular drusen >250 *μ*m, which are more dangerous [3]. Eyes diagnosed with the late-stage high-risk preangiogenic AMD, if left untreated, appear to have a significant risk for continual visual loss over the ensuing 12-month period [4].

Rheopheresis is an apparently safe and effective form of membrane differential filtration for the elimination of high molecular weight (HMW) proteins (i.e., fibrinogen, *α*_2_-macroglobulin, immunoglobulin M (IgM), thrombomodulin, and low-density lipoprotein (LDL) cholesterol) [5]. This method can normalise rheological parameters (the viscosity of blood and plasma as well as erythrocyte aggregability), can improve erythrocyte flexibility, and could lead to substantial improvement of visual functions in people suffering from AMD [6]. Consequently, this could improve blood flow in the choroid, which is reduced in the dry form of AMD [7].

The aim of our study was to determine the long-term effects of rheohaemapheresis on visual functions in advanced dry AMD and the development of drusenoid pigment epithelium detachment (DPED). The rheological effect of the therapy was monitored by a change in the levels of high-molecular substances in the blood (*α*_2_-macroglobulin, IgM, fibrinogen, and lipoprotein).

## 2. Group of Patiens and Controls

To date, we have treated 56 patients. In this study, we evaluated the long-term outcomes of patients with at least a 3.5-year follow up period. The group treated with rheohaemapheresis included 19 patients (4 men, 15 women) with the dry form of AMD, average age of 67.6 years (range 55–76 years). Patients were included in the study between March 2005 and November 2009, and follow up period was 42–84 months. At baseline, all patients had bilateral soft drusen in the macula while one patient had neovascular form of AMD in one eye at the time of entry into the study. Seventeen patients (30 eyes) also had DPED. During the follow up, neovascular AMD developed in two patients in one eye. Overall, we evaluated 35 eyes of 19 patients in the treatment group. In the control group, we evaluated results from 18 patients (2 males, 16 females) with the dry form of AMD and an average age of 72.8 years (range 64–81 years). Patients were enrolled in the study between June 2005 and November 2009, and follow up period was 42–84 months. At the start of the follow up, three patients had neovascular AMD in one eye. Seventeen patients (20 eyes) had DPED. During the follow up, neovascular AMD developed in six eyes of six patients. Overall, we evaluated 27 eyes of 18 patients in the control group. Eyes that suffered from neovascular AMD and/or developed neovascular AMD occurring during the follow up were not included in the subsequent evaluation. Exclusion criteria were retinal or choroidal disorders other than AMD, optic nerve disorders, glaucoma, conditions limiting the examination of the fundus, and acute bleeding in the studied eye. General exclusion criteria for rheohaemapheresis treatment were the usual exclusion criteria of extracorporeal circulation or therapeutic haemapheresis and the absence of peripheral veins suitable to establish an extracorporeal circuit. Patients with the late-stage, high-risk, preangiogenic form of AMD with soft drusen, confluent soft drusen, and DPED were recruited so that one patient was always assigned to rheohaemapheresis therapy and the second one joined the control group. This was repeated until the number of patients reached the target (limited by our technical and economic capabilities).

## 3. Methods

The cascade method of rheopheresis was used in the study group. Our modification of the cascade method (named rheohaemapheresis) was used for plasma separation. After plasma separation (blood cell separator, Cobe Spectra, Denver, CO, USA), the separated plasma was pumped through a rheofilter (Evaflux 4A, Kuraray, Osaka, Japan) to remove lipoproteins and other HMW rheologic factors (such as fibrinogen). We treated 1.5 volumes of plasma in one session. Every patient received the series of 8 procedures over 10 weeks.

An ophthalmological examination was performed in both groups at baseline and then every six months. In the treatment group, examination was also performed midcycle (after 4 weeks) and at the end of treatment (after 3 months). The reason for this was to check the immediate effect of treatment on retinal changes.

We examined the best-corrected visual acuity (BCVA) on Early Treatment Diabetic Retinopathy Study (ETDRS) charts under controlled illumination conditions. The examination distance was 2 m. The number of correct answers was noted and BCVA was considered as the row in which the patient detected at least three letters. The BCVA was converted into Snellen lines.

Fundus photography was performed using the digital fundus camera (Zeiss FF 450+IR; Zeiss, Jena, Germany). In our earlier study, we used the Reconstruct Programme for the geometric comparison of the DPED area [8]. However, the programme has some disadvantages. It is quite timeconsuming and the data for evaluating the measured area are in specially defined units that are not part of the SI system [9]. In this study, we used a Visupac system (Zeiss, Jena, Germany) in which the areas can be measured in mm^2^. The Visupac system was used to follow up the patients for 3.5-years from the start of rheohaemapheresis therapy. The acquired data were compared with the data from the untreated controls.

Fluorescein angiography (FA) was performed using the same digital fundus camera (Zeiss FF 450+IR) before evaluation and was repeated after 8 weeks and 1 year. Optical coherence tomography (OCT) was performed on the OCT Stratus (Zeiss, Jena, Germany) before evaluation to rule out cases of wet AMD and to confirm DPED. During the follow up, it was repeated every six months and whenever the occurrence of neovascular AMD or DPED was suspected. OCT and FA results were already partially published [8].

Electroretinography (ERG) was measured from the cornea with DTL fibre electrodes (Unimed Electrode Supplies, Surrey, UK). Reference and ground skin electrodes (gold cup electrodes) were attached to the ipsilateral temples and forehead, respectively. The pupils were fully dilated with 0.5% tropicamide and 10% phenylephrine for conventional photopic ERG and multifocal ERG (mfERG). The eyes were refracted for pattern (pERG) and mfERG. The examinations were performed before and up to 42 months after rheohaemapheresis in the treated patients and at the same time in the controls. We used the RETI-port plus mfERG system (Roland Consult GmbH, Brandenburg, Germany) for all types of ERG. Cone response and 30 Hz flicker and pattern ERG were registered under recording conditions according to the International Standard for Clinical Electroretinography (ISCEV) [10]. We analysed the amplitudes and implicit times of the a- and b-waves of the cone responses, the 30 Hz flicker responses, and P50 and N95 waves of pattern ERG [11].

The multifocal electroretinography (mfERG) stimulus was generated on a 20 inch ViewSonic monitor with a frame rate of 75 Hz and viewed at a distance of 30 cm. Sixty-one scaled hexagons stimulated the central 60° of the retina according to the ISCEV guidelines [10, 12]. Each element alternated between 160 cd/m^2^ (white flashes) and 1.4 cd/m^2^ (black flashes). The surrounding screen area was set to 80 cd/m^2^. A red cross was presented for fixation. Recordings were sampled at 1033 Hz, amplified by 200 K, and filtered with a frequency bandpass of 10–100 Hz. We evaluated the amplitudes and implicit times of the positive peak component (P1) of the first-order kernel analysis for five concentric rings centred on the fovea. Central element represented foveolar response (0°–1.8°), (1) ring (1.8°–7.0°), (2) ring (5°–13°), (3) ring (11°–22°), and (4) ring (17°–30°).

Important markers of the rheological efficacy of the procedures were evaluated 3 times (before the first, after the 4th, and after the last rheohaemapheresis). If necessary, further laboratory examinations were performed (e.g., after viral infections). Decreases of fibrinogen to under 0.7 g/L were considered critical. In these cases, the amount of filtered plasma was also decreased (from 1.5 body volumes to 1 volume).

The statistical comparison used nonparametric statistical tests (Kruskal-Wallis test, Mann-Whitney *U* test, and the chi-square approximation) and regression analysis.

The Institutional Ethics Committee approved the study protocol and the reported investigations were in accordance with the principles of the current version of the Declaration of Helsinki. An experienced eye specialist evaluated all eye findings of the study without knowing if the patient belonged to the control group or had been treated by rheohaemapheresis.

## 4. Results

### 4.1. BCVA

**Figure 1:**
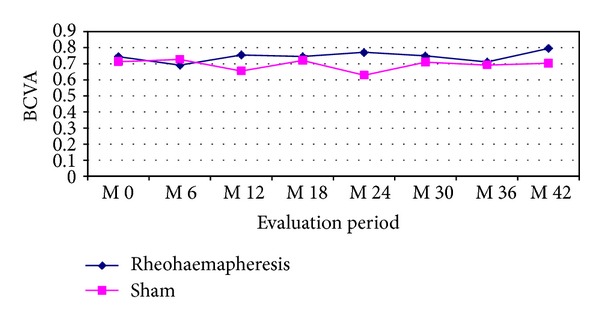
Development of BCVA during the follow up period in patients treated with rheohaemapheresis versus the control group.

In the group of patients who underwent rheohaemapheresis treatment, the average baseline BCVA of 0.74 (0.36–1.0) 95% CI was found. After 3.5 years of follow up, the average BCVA was 0.79 (0.41–1.0) 95% CI (*P* = 0.726). In the control group, the average baseline BCVA was 0.71 (0.15–1.0) 95% CI. After 3.5 years of follow up, the average BCVA decreased to 0.7 (0.32–0.87) 95% CI (*P* = 0.031). Development of BCVA during the follow up period in patients treated with rheohaemapheresis versus the control group is shown in Figure 1. Comparison of the final BCVA between the two groups was statistically significant at two years of follow up in favour of the treatment group (*P* = 0.028). Over the course of ongoing follow up, the difference in BCVA between the groups decreases and is no longer statistically significant 3.5 years after the beginning of the follow up (*P* = 0.125). However, it appears that BCVA tends to slightly improve over time in the treatment group, whereas it demonstrates a statistically significant decline in the control group.

### 4.2. DPED

**Figure 2:**
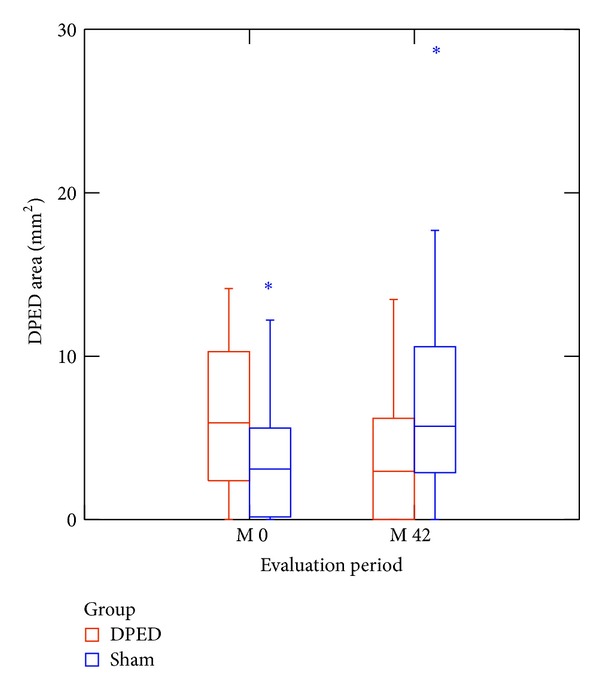
Changes in DPED areas between the two groups at the beginning and end of the follow up. The difference in DPED areas was statistically significant at the beginning and 3.5 years after the beginning of the follow up (*P* = 0.012 and *P* = 0.015, resp.).

The average DPED value at baseline in patients treated with rheohaemapheresis was 6.78 ± 3.79 mm^2^; after 3.5 years of follow up, it decreased to 4.13 ± 3.84 mm^2^ (*P* < 0.001). Patients in the control group had the average baseline DPED of 4.09 ± 3.48 mm^2^; after 3.5 years of follow up it increased to 6.69 ± 4.2 mm^2^ (*P* = 0.001). A graphical comparison of changes in DPED areas between the two groups at the beginning and end of the follow up is shown in Figure 2.

### 4.3. ERG Pattern

Regression analysis confirmed a gradual, statistically significant (*P* = 0.022) increase in values of positive wave P50 in treated patients. After 3.5 years, the amplitude of their P50 waves increased by approximately 16%. In contrast, the amplitude of the P50 wave in the control group only insignificantly varied throughout the whole follow up period and at the end of follow up and it was significantly lower in comparison with that in treated patients (*P* = 0.024). Changes in the amplitude of negative wave N95 and latency of both P50 and N95 waves did not reach statistical significance over time in either group of patients and mutually differed insignificantly. Despite the above, the N95 wave also appeared to have a tendency to slightly increase in amplitude in the group of RHF-treated patients (*P* = 0.055).

### 4.4. Electroretinography (ERG)

Using regression analysis, we found relatively stable amplitudes of photopic responses of treated patients; in contrast, there was a trend towards a significant (*P* = 0.039 for b-wave response of cone and *P* = 0.019 for 30 Hz flicker) decrease of photopic activity in the control group of approximately about 11% for cone response and 13% for 30 Hz flicker after 3.5 years. The differences in the amplitudes were insignificant between the groups of patients. The implicit times of the majority of responses increased significantly in both groups (*P* < 0.001); moreover, they were significantly longer in the controls (*P* < 0.001).

### 4.5. Multifocal Electroretinography (mfERG)

Regression analysis confirmed an insignificant fluctuation of foveal activity amplitudes and responses in the most peripheral area of the retina in both groups of patients. In the parafoveal and paramacular regions in eccentricity between 1.8° and 13°, we found a trend of a gradual, albeit insignificant, increase in the activity up to 2.5 years in treated patients, and then the activity in this area remained almost unchanged. In the control group, the activity in the same region was originally only slightly increased for the first six months but then started to gradually decrease. In terms of examination, the differences were statistically insignificant for a majority of patients between the two groups. However, the activity in the region at eccentricities of 5° and 13° was significantly higher in treated patients (*P* = 0.04 and *P* = 0.01, resp.) at follow ups at 1.5 and 2.5 years. The implicit times of the majority of responses increased during 3.5 years in all patients. At the baseline, they were significantly longer in the controls (*P* values ranging from <0.05 to <0.01), with the exception of the foveolar response. At 3.5 years, the foveolar response was significantly longer in the control group (*P* = 0.035).

Central retinal activity (mainly parafoveal activity between 1.8° and 7° of eccentricity or even paramacular activity between 5° and 13°) increased in the treated patients with an early decrease or complete disappearance of the DPED area. In contrast with patients with long lasting or persistent DPED, retinal activity and visual acuity do not improve or may have even reduced.

### 4.6. The Incidence of CNV

During the follow up, we determined the occurrence of CNV in two eyes in the group treated with rheohaemapheresis and six eyes in the control group (Table 1). Comparison between both groups was statistically insignificant.

### 4.7. Rheological Markers

Evaluation of blood biochemical results before the procedures and immediately after the procedures showed statistically significant changes (Table 2). These changes were, however, not clinically significant and did not put patients at risk. Basic haematological parameters were also monitored, including blood count and platelet parameters. Results showed that basic blood count parameters (haemoglobin, hematocrit, and leukocytes) are not significantly affected by the therapeutic procedures from the clinical point of view, although statistical significance can be determined (Table 3). We also monitored markers of rheohaemapheresis efficacy (*α*_2_-macroglobulin, IgM, fibrinogen, and lipoproteins and the resulting effect on the viscosity of blood and plasma). The effect of therapy was to reduce the defined range of high-molecular substances. Results are illustrated in Table 4; it is apparent that the therapeutic procedures are very effective and result in a reduction of *α*_2_-macroglobulin by 58%, fibrinogen by 66%, IgM by approximately 65%, LDL cholesterol by 70%, and apolipoprotein B by 72%; an additional risk factor decreased as well—lipoprotein(a) by approximately 59%. The decrease of the above factors ultimately affected the rheological conditions: whole blood viscosity decreased by 15% and plasma viscosity by 12%. Furthermore, we monitored specific markers which might indicate an effect on the inflammatory activity (interleukin-(IL-) 10), atherosclerotic activity (soluble form of CD30 (sCD30), high sensitivity C-reactive protein (hsCRP)), an effect on the markers of cellular immunity (monocyte chemotactic peptide-(MCP-) 1, sCD30), and adhesion molecules (such as selectins) (Table 5).

## 5. Discussion

In order to maintain the best BCVA and reduce the risk of migration into the terminal stages of the disease in the form of geographic atrophy or even wet AMD, which are associated with a significant decrease of visual acuity, patients with dry AMD and soft drusen were indicated for rheohaemapheresis treatment. During the long-term follow up, we demonstrated BCVA improvement in patients with advanced dry AMD, similarly to authors of the MAC-I study from the University of Cologne, MAC-II study from the University of Frankfurt, and MAC-III study from the University of Hamburg [5]. In comparison with the results of a multicentre, randomised, double-blind MIRA-1 study, we demonstrated improved BCVA in the group of treated patients and statistically significant deterioration in the control group of patients in the long term. Complex assessment of the MIRA-1 study was, however, impacted by incompliance with the criteria for inclusion into the treatment group and subsequent analysis after excluding patients who did not meet the inclusion criteria and confirmed the effects of rheohaemapheresis after one-year follow up [13, 14]. Unlike the MIRA-1 study, our study was not double blind, because our ethics committee would not allow extracorporeal circulation in a double-blind study setup and because rheohaemapheresis can, in general, have serious side effects (although these were not observed in our study).

In our previous study, BCVA was stable in treated patients up to 18 months after the initiation of RH, whereas BCVA decreased significantly at 18 months of follow up in controls [8]. Our long term results confirmed this observation and showed a tendency for the visual acuity of patients to improve in the long term, although this was not statistically significant. The decrease of BCVA in the treatment group can be explained by delayed improvement of microcirculation in the retina that demonstrably occurs after rheohaemapheresis. The gradual deterioration of BCVA in the control group corresponds to the natural progression of the disease which was expressed also by the higher share of newly developed neovascular AMD in the control group.

Our findings of increased amplitudes of pERG responses, significant for the P50 waves and insignificant for the N95 waves, indicated the positive effect of rheohaemapheresis treatment on the central regions of the retina and ganglion cells in patients after rheohaemapheresis. We did not find any assessment of pERG in patients with AMD treated with rheohaemapheresis in the literature. In this study, we found a stable photopic activity of treated patients compared to its significant decrease in the control group. The evaluation of the electrical activity of the retina in patients with AMD treated with rheohaemapheresis is provided by only a few authors. They describe either insignificant changes of photopic responses [8] or increased cone response after 2.5 years [15]. Rencová et al. found significantly increased central retinal activity in the paramacular region in eccentricity between 5° and 13° after rheohaemapheresis [8]. Blaha et al. found improvement of responses in the boundary region between 11° and 22° of eccentricity in the treated patients [15]. In our study, the central retinal activity and BCVA increased in the treated patients with an early decrease or complete disappearance of the DPED area. In contrast in the patients with long lasting or persistent DPED, retinal activity and visual acuity may be even paradoxically reduced due to degenerative changes of the outer neuroretinal layers, as demonstrated by OCT [16].

One goal of rheohaemapheresis is to achieve resorption of soft drusen. Initial results were presented in our previous study [8] in which we also demonstrated the influence of rheohaemapheresis on the reduction of size or disappearance of DPED. If the soft drusen progress into the DPED, which is the normal development of AMD, this pathological change gradually increases and shows no tendency for resorption. If resorption occurs after few years, it is usually replaced by atrophy of the retinal pigment epithelium (RPE), most often in the form of geographic atrophy. This terminal stage of dry AMD is obviously associated with a significant decrease in BCVA [17]. Our results show that the DPED area decreases after rheohaemapheresis and, in the long term, large geographic atrophy of RPE does not develop. Khanifar et al. mention the risk of transformation of drusen merging in the DPED as well as individual high drusen into the wet form of AMD [18]. Sikorski et al. use spectral OCT with high resolution power and point to the possibility of fluid accumulation under the junction of internal and external segments of photoreceptors (IS/OS) in the recesses between adjacent, already partially confluent drusen. This fluid increases the distance between the RPE and the thin IS/OS photoreceptor junction. The local presence of fluid under the neuroepithelial layer of retina surprisingly does not yet indicate the presence of submacular neovascularization and thus the wet form of AMD [16]. This is how we explain the statistically insignificant decrease in visual acuity and insignificant reduction of multifocal ERG values in cases where the treatment results in the reattachment of DPED without development of RPE atrophy. RPE atrophy following reattachment of DPED after rheohaemapheresis may occur, but due to its small size it was not associated with a significant decrease in BCVA of the treated patients. According to Sikorski et al., defect in the thin IS/OS layer adjacent to the RPE can occur on top of a particularly prominent druse or DPED and can be associated with penetration of fluid, which is a sign of transformation into the wet form of AMD [16]. This occurred in two eyes of our treated patients several years after the rheohaemapheresis. Compared to that in the control group, the frequency of transformation into the neovascular AMD is lower, although not statistically significant due to this small size of the group. Unlike in the control group, where we observed the transformation into the neovascular AMD in one eye as early as six months after the start of follow up, rheohaemapheresis delays disease progression. This finding leads us to the assumption that in order to achieve maximum effect it will probably be necessary to repeat rheohaemapheresis 1.5–2 years after the last cycle. This assumption is also supported by the results of mfERG, which remain unchanged between the 30th and 42nd months despite their original slight improvement.

Evaluation of biochemical and haematological parameters before and immediately after rheohaemapheresis showed values which, although statistically important, were clinically insignificant. As far as the decrease in values is considered, it could be partially attributed to dilution changes (after the procedure, there is a small, approximately 5–10%, blood dilution). A certain increase in the number of leukocytes, although entirely within normal range, is generally observed after these procedures are performed in any clinical indication. As is common for any other haemapheresis procedure, the number of haemoglobin and platelets decreases slightly, but the decrease is lower when using current advanced separators (as in this study) compared to that when using the older separator types. Procedures are therefore safe as far as their impact on the blood count is considered. The decrease of *α*_2_-macroglobulin, IgM, fibrinogen, and lipoprotein influences rheological conditions: whole blood viscosity decreased by 15% and plasma viscosity by 12%. The result is undoubtedly an improved microcirculation flow, which is also a basic prerequisite for increased flow in the choroid and improved retinal metabolism.

We have demonstrated that some of the above evaluated indicators suggest a decline in the activity of vascular endothelium or inflammatory changes in our patients after rheohaemapheresis treatment. Improved tissue perfusion after rheohaemapheresis may be also associated with the decrease in the levels of soluble forms of selectin adhesion molecules E-selectin and P-selectin in the peripheral blood [19]. These molecules, which regulate the initial stages of leukocyte and platelet adhesion, are stored in intracellular granules from which they are rapidly transported to the surface after activation. E-selectin is located exclusively in the Weibel-Palade bodies of endothelial cells, and P-selectin is stored in both the alpha-granules of platelets and the Weibel-Palade bodies of endothelial cells. It was, however, demonstrated that the soluble P-selectin (sP-selectin) in peripheral blood is almost exclusively released from the alpha-granules of platelets [20]. Measurements of sP-selectin and soluble E-selectin (sE-selectin) can be probably used to evaluate the effect of procedures on the endothelial lining of blood vessels. In our group, sP-selectin as well as endoglin levels decreased after treatment. Levels of sE-selectin were monitored in early stages of the study, but we did not obtain any positive results, because its levels did not change and this parameter was therefore no longer monitored during the ongoing study.

We previously evaluated MCP-1 during our earlier research of rheohaemapheresis use due to the significant importance of macrophages in the microcirculation and we found a significant decrease in patients with familial hypercholesterolemia [19, 21]. This study demonstrated its decrease in patients with AMD. As stated in the literature, this may be a significant factor, indicating efficacy of the impact on activity of the inflammatory process or atherosclerosis [5, 19]. The mechanism of the inflammatory process modulation by rheohaemapheresis in the pathogenesis of AMD could also be documented by a significant decrease in inflammatory markers IL-10, IgM, or CD30 and sCD40L in case of atherosclerotic mechanism, respectively.

## 6. Conclusion

In our study, we demonstrated the long-term effects of rheohaemapheresis on the dry form of AMD at the stage of soft confluent drusen and DPED. During our 3.5-year follow up, BCVA deteriorated in the control group of patients without treatment and this deterioration was statistically significant. BCVA in patients treated with rheohaemapheresis improved. BCVA improvement in the treatment group was accompanied by improvement in the function of photoreceptors in the central region of retina and ganglion cells which could be demonstrated on pERG and mfERG. Functional changes were accompanied by structural changes such as reduction of the DPED area after rheohaemapheresis and progression of findings in patients in the control group. The effects of rheohaemapheresis could be explained by improved blood flow to the retina and choroid due to the reduced viscosity of whole blood and plasma. Further research, motivated by our findings of the decrease in some markers of endothelial activity, inflammatory markers, and atherosclerotic markers, could help us explain the additional effects of rheohaemapheresis and contribute to the improved knowledge of AMD etiopathogenesis.

## Figures and Tables

**Table 1:** The incidence of CNV during the follow up.

	6 months	1 year	2 years	3 years	4 years	5 years	6 years
Rheohaemapheresis	—	—	I	I	—	—	—
Sham	I	I	I	I	I	—	I

I: one eye.

**Table 2:** The blood biochemical results before the procedures and immediately after the procedures.

Blood biochemical markers	Before rheohaemapheresis		After rheohaemapheresis		Relative change in value %	*P*
	Mean	SD	Mean	SD		
Glycemia	5,76	1,50	6,35	1,55	10,24	<0,001
Sodium	139,33	10,03	141,52	3,54	1,57	<0,001
Potassium	3,96	0,26	3,83	0,30	−3,28	<0,001
Chloride	103,72	3,39	105,39	3,79	1,61	<0,001
Calcium	2,22	0,12	2,13	0,23	−4,05	<0,001
Urea	5,66	1,57	5,09	1,46	−10,07	0,002
Creatinine	76,62	16,32	67,25	15,70	−12,23	<0,001
Uric acid	323,72	97,84	293,22	94,15	−9,42	0,002

SD: standard deviation.

**Table 3:** Haematological parameters before and after rheohaemapheresis.

Parameters	Before rheohaemapheresis		After rheohaemapheresis		% of difference	*P*
	Mean	SD	Mean	SD		
Leucocytes (×10^9^/L)	6,68	2,16	7,35	2,17	10,03	<0,001
Haemoglobin (g/L)	134,77	13,50	128,98	15,17	−4,30	<0,001
Hematocrit	0,40	0,04	0,38	0,04	−5,00	<0,001
MCV (fl)	89,61	9,51	90,39	4,72	0,87	0,786
Thrombocytes (×10^9^/L)	225,44	49,61	216,08	42,93	−4,15	0,032
MPV (fl)	10,60	1,03	10,50	0,89	−0,94	0,421
PDW (%)	13,85	2,44	13,04	2,20	−5,85	<0,001

SD: standard deviation.

MCV: mean cell volume.

MPV: mean platelet volume.

PDW: platelet distribution width.

**Table 4:** Indicators of rheological effectivity.

Followed parameter	Mean	SD	Mean	SD	% of difference	P
Immunoglobulin M (g/L)	0,85	0,59	0,30	0,35	−64,50	<0,001
*α*_2_-macroglobulin (mg/dL)	136,19	56,18	56,99	35,36	−58,16	<0,001
Total cholesterol (mmol/L)	4,33	1,25	1,76	0,49	−59,28	<0,001
LDL cholesterol (mmol/L)	2,45	1,02	0,74	0,37	−69,85	<0,001
Lipoprotein (a) (g/L)	0,23	0,31	0,10	0,19	−58,92	<0,001
Apolipoprotein B (g/L)	0,71	0,26	0,20	0,15	−72,09	<0,001
Fibrinogen (g/L)	2,96	0,68	1,02	0,29	−65,68	<0,001
Blood viscosity (mPa·s)	6,24	1,29	5,31	0,92	−14,89	<0,001
Plasma viscosity (mPa·s)	2,06	0,35	1,81	0,42	−11,96	<0,001

SD: standard deviation.

LDL: low-density lipoprotein.

**Table 5:** Specific haematological markers.

Followed parameter	Before procedure			After procedure				
	Mean	SD	Median	Mean	SD	Median	Statistical significance	% of difference
hsCRP	3,56	5,89	2,5	2,74	4,21	2,09	<0,001	16,40
IgM	0,816	0,554	0,674	0,3	0,354	0,18	<0,001	73,29
IL-10	59,01	25,65	56,4	53,82	23,85	54,65	0,001	3,10
MCP-1	275,1	78,31	256,4	265,7	275,4	241,2	<0,001	5,93
sCD30	46,1	21,86	43,6	37,51	19,39	33,2	<0,001	23,85
sCD40L	5453	2182	5007	5018	2202	457,6	<0,001	90,86
sP-selectin	150,6	54,61	147,3	133,1	55,74	125,3	<0,001	14,94
Endoglin	6,34	1,57	6,5	5,43	1,58	5,2	<0,001	20,00

SD: standard deviation.

hsCRP: high sensitivity C-reactive protein.

IgM: immunoglobulin M.

IL-10: interleukin-10.

MCP-1: monocyte chemotactic peptide-1.

sCD30: soluble CD30.

sCD40L: soluble CD40L.

sP-selectin: soluble P-selectin.
